# Alterations in brain network connectivity and subjective experience induced by psychedelics: a scoping review

**DOI:** 10.3389/fpsyt.2024.1386321

**Published:** 2024-05-14

**Authors:** Zijia Yu, Lisa Burback, Olga Winkler, Lujie Xu, Liz Dennett, Eric Vermetten, Andrew Greenshaw, Xin-Min Li, Michaela Milne, Fei Wang, Bo Cao, Ian R. Winship, Yanbo Zhang, Allen W. Chan

**Affiliations:** ^1^ Department of Psychiatry, University of Alberta, Edmonton, AB, Canada; ^2^ Sperber Health Sciences Library, University of Alberta, Edmonton, AB, Canada; ^3^ Department of Psychiatry, Leiden University Medical Centre, Leiden, Netherlands; ^4^ Neuroscience and Mental Health Institute, University of Alberta, Edmonton, AB, Canada; ^5^ Institute of Human Nutrition at the Vagelos College of Physicians and Surgeons, Columbia University, New York, NY, United States; ^6^ Nanjing Medical University Affiliated Brain Hospital, Nanjing, Jiangsu, China

**Keywords:** psychedelics, functional connectivity, LSD, psilocybin, MDMA, ayahuasca, subjective effects

## Abstract

Intense interest surrounds current research on psychedelics, particularly regarding their potential in treating mental health disorders. Various studies suggest a link between the subjective effects produced by psychedelics and their therapeutic efficacy. Neuroimaging evidence indicates an association of changes in brain functional connectivity with the subjective effects of psychedelics. We conducted a review focusing on psychedelics and brain functional connectivity. The review focused on four psychedelic drugs: ayahuasca, psilocybin and LSD, and the entactogen MDMA. We conducted searches in databases of MEDLINE, Embase, APA PsycInfo and Scopus from inception to Jun 2023 by keywords related to functional connectivity and psychedelics. Using the PRISMA framework, we selected 24 articles from an initial pool of 492 for analysis. This scoping review and analysis investigated the effects of psychedelics on subjective experiences and brain functional connectivity in healthy individuals. The studies quantified subjective effects through psychometric scales, revealing significant experiences of altered consciousness, mood elevation, and mystical experiences induced by psychedelics. Neuroimaging results indicated alterations in the functional connectivity of psychedelics, with consistent findings across substances of decreased connectivity within the default mode network and increased sensory and thalamocortical connectivity. Correlations between these neurophysiological changes and subjective experiences were noted, suggesting a brain network basis of the psychedelics’ neuropsychological impact. While the result of the review provides a potential neural mechanism of the subjective effects of psychedelics, direct clinical evidence is needed to advance their clinical outcomes. Our research serves as a foundation for further exploration of the therapeutic potential of psychedelics.

## Introduction

In the 1950s and 1960s, Western science recognized that psychedelic substances like lysergic acid diethylamide (LSD), psilocybin, ayahuasca (main psychoactive ingredient DMT), and 3,4-methylenedioxymethamphetamine (MDMA), may alter human perception, thought and emotion, and psychedelic drugs were explored for their potential to treat psychiatric disorders (1). Use of these substances was halted following conservative societal rejection through the implementation of drug control conventions and narcotics laws under United Nations treaties [see (2)]. There has been a recent resurgence of interest in psychedelic drugs and their therapeutic potential for the treatment of mental disorders (3–6). For example, clinical trial evidence is now available that indicates the likely efficacy of psychedelic-assisted psychotherapy in treatment-resistant depression (TRD) and post-traumatic stress disorder (PTSD) (6). Specifically, research has explored MDMA’s therapeutic potential in treating PTSD. MDMA, known by its alternative name ‘ecstasy’ or ‘entactogen,’ exerts distinct effects on the brain compared to classical psychedelics like LSD or psilocybin. Notably, it enhances the release of hormones such as oxytocin, often referred to as the ‘love hormone’ due to its role in social bonding and trust (7). For instance, the ability of MDMA to enhance positive mood and empathy may assist individuals with PTSD in revisiting traumatic memories with reduced fear and avoidance. This emotional engagement is pivotal, as it enables the processing and integration of traumatic experiences during psychotherapy.

Subjective effects induced by psychedelics are believed to have a significant correlation with their clinical therapeutic outcomes. Psilocybin-induced ‘high oceanic self-boundlessness (OSB)’ and low dread of ego dissolution (DED) in TRD patients are associated with the prediction of clinical outcomes (8). Open-label studies on psilocybin-assisted psychotherapy for tobacco addiction and alcoholism have identified notable correlations between mystical experiences and enhanced therapeutic results (9). This has given rise to the question of whether specific subjective effects are necessary for the therapeutic efficacy of psychedelics. It is critically important to understand the mechanisms through which psychedelics induce subjective effects to elucidate the necessary aspects of therapeutic action in this context. By understanding the neural and psychological processes underpinning these effects, we can gain valuable insights into the therapeutic potential of psychedelics and optimize their use as medicines for treatment of psychiatric disorders (10) (5). Such understanding may open up new strategies for fundamental research and drug development for treatment of mental disorders.

In this review, we focus on three classical psychedelics: LSD, psilocybin, and ayahuasca, as well as one non-classical psychedelic: MDMA. The distinction between classical and non-classical psychedelics is typically based on their chemical structure and pharmacological effects. Classical psychedelics generally have an indole structure and occur in nature, primarily acting on 5-hydroxytryptamine (5-HT) 2A receptors. In contrast, non-classical psychedelics have a non-tryptamine structure with distinct binding sites that differ from classic psychedelics (11). LSD exhibits high binding affinities to various receptors, including 5-HT receptors (5-HT1A, 5-HT2A, 5-HT2C), dopamine D2 receptors, and α2 adrenergic receptors. It also has low-affinity binding to other dopamine receptors (12). Psilocybin and its active metabolite psilocin (13) bind to several receptors, including 5-HT receptors (5-HT2A, 5-HT2B, 5-HT2C), dopamine receptors, histamine receptors (14), and interleukin receptors (15). Ayahuasca, a preparation derived from a mixture of two Amazonian plants, contains N, N-dimethyltryptamine (DMT), its key hallucinogenic component (16). DMT’s psychobiological effects primarily stem from activating 5-HT receptors (5-HT2A, 5-HT1A, and 5-HT2C) and norepinephrine (NE) (17). The non-classical psychedelic MDMA acts on multiple neurotransmitter systems, binding not only to 5-HT receptors, dopamine receptors, and adrenergic receptors (specifically, alpha-2 receptors) (17), but also significantly influencing oxytocin levels (18).

Exploring the effects of psychedelics on the brain’s functional connectivity has garnered significant attention in recent research. Typically assessed using functional magnetic resonance imaging (fMRI), electroencephalography (EEG), and magnetic electroencephalography (MEG) (19) (20), functional connectivity pertains to the statistical interdependence or correlation of neuronal activity patterns between distinct brain regions (21). The essence of functional connectivity lies in the idea that the correlated regions are functionally interrelated or dependent on each other.

Research into the impact of psychedelics on functional connectivity has revealed consistent findings. For instance, LSD reduced connectivity within the default mode network (DMN), which is associated with emotional processing, self-referential mental activity, and memory recollection, encompassing regions such as ventral medial prefrontal cortex (vmPFC), dorsal mPFC, posterior cingulate cortex (PCC), adjacent precuneus, and lateral parietal cortex (22–25). Similarly, psilocybin administration decreased functional connectivity between the mPFC and PCC within the DMN in healthy participants (26). The DMN, typically active when the mind is in a restful state and not engaged with the external environment, is often linked to self-referential thinking (27). Interestingly, while TRD patients exhibited increased functional connectivity within the DMN (28), those with MDD displayed the opposite trend (29). Such DMN dysregulation in MDD may correlate with the ruminative thought patterns observed in these patients (30). Another psychedelic, DMT, may decrease connectivity within the PCC/precuneus, a component of the DMN. Intriguingly, DMT also increases the connectivity between the DMN and the salience network (SN), along with increasing connectivity within the ACC of the SN (31–33). The SN, consisting of regions like the amygdala (AMG), anterior insula, and dorsal ACC (dACC), plays a pivotal role in assessing the importance of both internal and external stimuli (34). It’s noteworthy that PTSD patients have exhibited persistent hyperconnectivity within the SN (35), which may underpin their intensified responses to perceived threats (36).

The underlying mechanisms by which psychedelics exert therapeutic effects in the context of mental disorders are not yet fully understood. While a significant body of research now describes the subjective effects of these substances, the possible direct relationship to therapeutic outcomes needs further elucidation. For instance, Roseman found that specific subjective experiences following administration of psychedelics, such as high OSB and low DED, could predict the reduction of severity of depressive symptoms in TRD patients (8), as measured by the 16-item Quick Inventory of Depressive Symptoms (QIDS-SR). OSB refers to feelings of unity and positive emotions often associated with a form of ego dissolution, and DED refers to an unpleasant state of anxiety marked by thought disorder, loss of autonomy, and reduced self-control (37). Combined with lower DED and higher OSB in TRD patients, the result emphasizes that anxiety levels and letting go of mental defense is important in the therapeutic effects of psychedelics (8). In addition, mystical experience is also highly correlated with the improvements in clinical outcomes in TRD patients and patients with alcohol dependence (38).

The therapeutic efficacy of psychedelics and its correlation with subjective effects remains a topic of debate. Some, like Yaden, posit that subjective experiences are necessary for the positive therapeutic effects of psychedelics by emphasizing evidence that psilocybin-occasioned mystical experiences are important in the treatment of tobacco addiction (39). Yaden further emphasizes the value of other subjective effects, such as profound insights and emotional breakthroughs, in the potentially therapeutic effects of psychedelics (40). Olson, on the other hand, indicates that the subjective effects are not a necessary condition for psychedelics to produce lasting therapeutic results since there are non-hallucinogenic compounds known to enhance neuroplasticity and have antidepressant, anxiolytic, and anti-addictive effects. However, Olson also supports that these subjective experiences might be important in maximizing therapeutic outcomes (39), especially considering that mystical experiences consistently correlate with therapeutic efficacy, potentially implicating psychological mechanisms or a potent placebo response (41). Based on the balance of evidence and opinion, understanding the underlying mechanisms of these subjective effects may offer insights into potential response predictors and modulators, enabling the refinement of therapeutic protocols and potentially improving patient outcomes.

Overall, this review seeks to examine the link between the subjective effects of psychedelics and the alterations in functional connectivity they induce. By understanding this relationship, we may shed light on the deeper mechanisms through which psychedelics affect the human brain and psyche, potentially paving the way for more targeted and effective future therapeutic interventions.

## Methods

This scoping review followed the Preferred Reporting Items for Systematic Reviews and Meta-Analyses – Scoping Review (PRISMA- ScR) methodology developed by the Joanna Briggs Institute (42) and no formal review protocol has been registered for the review. We conducted searches in databases of MEDLINE, Embase, APA PsycInfo and Scopus from inception to June 2023 by keywords related to functional connectivity and psychedelics. Details of the search strategy are provided in the **Supplementary Materials** section.

We specifically focused on peer-reviewed clinical studies involving MDMA, LSD, ayahuasca, or psilocybin and the use of resting-state fMRI data to measure functional connectivity. Excluded are non-clinical studies, ketamine, or cannabis-related studies, those reporting on effective connectivity, directed connectivity, or dynamic functional connectivity without mentioning functional connectivity, and studies using non-fMRI data or a task-positive fMRI study design. This review did not consider conference papers, preprints, case reports, and review papers to enhance result reliability and reduce heterogeneity.

Two reviewers were responsible for the overall screening process, including the title and abstract screening and the full-text screening, with a third reviewer engaged in resolving any differences of opinion on inclusion that could not be resolved through discussion. In cases of disagreement the third reviewer had an independent deciding vote. Data from the included studies were extracted using Covidence in accordance with the participants, intervention, comparison, and outcomes framework (43), which allowed us to consistently examine and categorize the key elements of each study. For this review, the ‘outcomes’ component was divided into functional connectivity and subjective effects.

## Results

**Figure 1:**
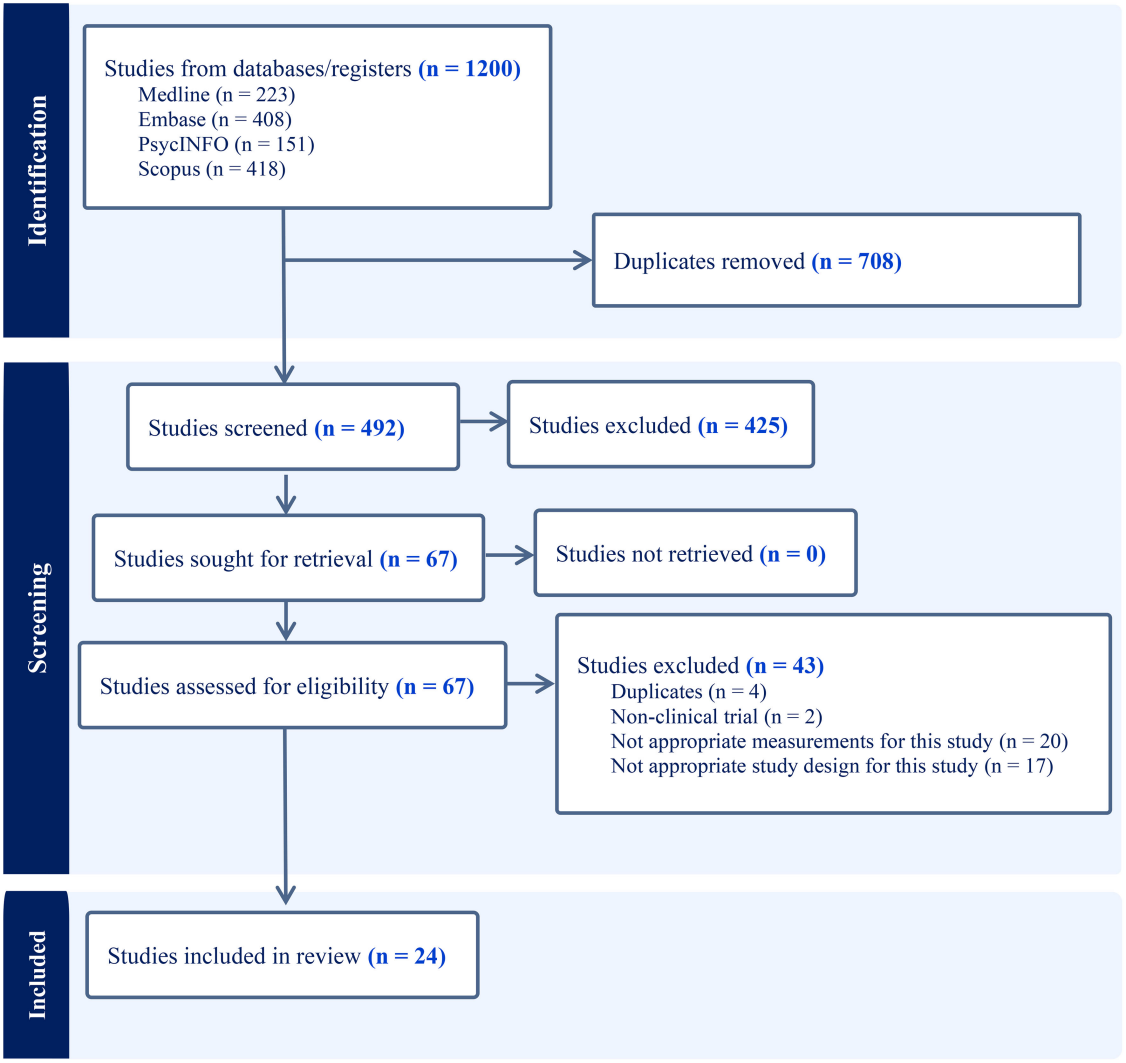
The flow chart of PRISMA.

As the PRISMA flow chart (**Figure 1**) depicts, we imported 1200 articles from four databases, out of which 708 duplicates were eliminated, leaving us with 492 articles for title and abstract screening. Applying our inclusion and exclusion criteria, 67 articles progressed to the full-text screening phase. Of those, only 24 articles met final inclusion standards. The details of the search results are shown in **Table 1**. Participant characteristics and study findings are summarized in **Tables 2**, **3**.

**Table 1:** Summary of article selection and characteristics for psychedelic study review.

Criteria	Number
Total articles after full-text screening	67
Non-clinical trials	2
Duplicates	4
Experimental designs not meeting criteria	17
Results not relevant for extraction	20
Articles included in the review	24
Original studies	18
Secondary analyses	6^*^
Original studies with placebo control	14
Original studies with dose-response trials	1
Original studies with pre-post studies	3
Original studies with randomized studies	10
Original studies with double-blind experiments	9
Original studies with single-blind experiments	3
Original studies with crossover study designs	12
Total participants	406
Healthy participants	382
Healthy participants with history of drug/stimulant use	Most
Healthy participants experienced meditation	53
TRD or PTSD patients	24
Studies involving LSD	8
Studies involving ayahuasca	3
Studies involving psilocybin	9
Studies involving MDMA	5
Studies involving both LSD and MDMA	1

^*^Details of 6 secondary analyses are as follows: Psilocybin studies (44) and (45) reanalyze data from (26); LSD study (24) reanalyzed data from (46); studies (22) and (23) share a sample; MDMA study (47) reanalyzed data from (48).

**Table 2:** Summary of study characteristics, subjective effects measurements, and significant findings.

Substance	Subjective Effects Scales	Subscales of the Scales	Significant Findings
Ayahuasca (DMT)	YMRS, HRS	HRS evaluates subjective effects of hallucinogens, including somesthesis, affect, perception, cognition, volition, and intensity.	YMRS: Significant changes in 5 out of 11 items, including elevated mood, increased motor activity-energy, speech (rate and amount), language-thought disorder and content (32). HRS: Increase in ayahuasca-induced effects for all subscales except volition in one study (33); significant effects including volition in another (31).
LSD	5D-ASC, 11D-ASC, MEQ, VAS	5D-ASC: OSB, DED, VRS, AUA, VIR. 11D-ASC: experience of unity, spiritual experience, blissful state, insightfulness, disembodiment, impaired control and cognition, anxiety, complex imagery, elementary imagery, audio-visual synesthesia, changed meaning of percepts. VAS: intensity, simple hallucinations, complex hallucinations, emotional arousal, positive mood, ego-dissolution. MEQ: mystical experience, positive mood, transcendence of time and space, and ineffability.	5D-ASC: Significant increases in all major dimensions (24, 49–51); LSD did not significantly alter ratings on the 5D-ASC (52); 11D-ASC: Dose-dependent changes in blissful state, insightfulness, and changed meaning of percepts (24). Increase in ten of the eleven factors, except “anxiety” (23, 53). MEQ: mystical experiences were reported (24). VAS: All subscales are significant increased after LSD treatment (23).
Psilocybin	5D-ASC, MEQ, VAS, MAAS	MEQ: pure being and pure awareness, fusion of your personal self into larger whole, sense of reverence or sacredness, timelessness (lack of sense of space/time), ineffability (inability to explain experience with words), feelings of joy, feelings of peace and tranquility. MAAS: mindful attention awareness scale, 15 items.	5D-ASC: significant increases in all scales (54); changes only in OSB, VRS and VIR (55). MEQ: increased overall drug effect strength and ratings of mystical experience (56). A wide range of emotions and mystical experiences, such as joy, letting go, nowness, and reverence (26). Subjective effects like “equanimity”, pure being and pure awareness, feelings of joy, and an aggregate measure of mystical experience and increases in feeling of joy (57). VAS: changes in perception, imagination, time perception, and overall dreamlike quality (26). MAAS: Increased trait openness and mindfulness (44).
MDMA	5D-ASC, VAS, AMRS, MEQ, 29-items measurement	AMRS: 11 items	5D-ASC: no significant different changes of ratings, with the exception of “blissful state” (50). 29-items measurement: most volunteers reported positive mood effects, and items like “I felt amazing” referring to aspects of positive mood were among the highest scored (58).

OSB, Oceanic Boundlessness; DED, Dread of Ego Dissolution; QIDS-SR, Quick Inventory of Depressive Symptoms; 5D-ASC, 5 Dimensions Altered States of Consciousness; 11D-ASC, 11 Dimensions Altered States of Consciousness; HAM-A, Hamilton Anxiety Rating Scale; HADS, Hospital Anxiety and Depression Scale; YMRS, Young Mania Rating Scale; HRS, Hallucinogen Rating Scale; AUA, auditory alterations; VIR, vigilance reduction; VRS, Visionary Restructuralization; VigRe, Vigilance Reduction; MEQ, Mystical Experience Questionnaire, VAS, Visual Analog Scale; AMRS, Adjective Mood Rating Scale; ARCI, Addiction Research Center Inventory; CAPS-IV, Clinician-Administered PTSD Scale for DSM-IV; MAAS, Mindful attention awareness scale.

**Table 3:** Overview of studies on the effect of psychedelics on functional connectivity and subjective effects.

Psychedelics	Male/ Female	Age (mean ± SD]	Dose	Participants	Study design	Resting-state functional connectivity	Correlation between functional connectivity and subjective effects
Ayahuasca (DMT) (32)	5/5	29	0.8 mg/kg DMT, po.	Ten healthy volunteers have at least 5 years of regular (twice a month) Ayahuasca	Pre- and post-test design.	Ayahuasca decreased functional connectivity within the PCC/Precuneus and functional connectivity of PCC-DMN.	Decreased functional connectivity within the PCC/Precuneus was correlated with the increased YMRS scores.
Ayahuasca (31)	12/10	30.8 ± 8.4 (Aya group) 31 ± 10.5 (placebo group)	0.36 mg/kg N, N-DMT, po.	All participants were healthy and naïve to ayahuasca.	Parallel design, placebo-controlled design.	Ayahuasca increased functional connectivity within ACC, and between SN and DMN; It decreased functional connectivity within PCC.	Increased functional connectivity within SN correlates with increased somesthesia on HRS, decreased functional connectivity within DMN correlates with increased volition on HRS, and increased SN-DMN connectivity correlates with increased affect on HRS.
Ayahuasca (DMT) (33)	10/6	38.9 ± 7.8	0.64 mg/kg DMT, po.	All participants received LSD at least once.	Pre- and post-test design.	Ayahuasca increased functional connectivity of PCC-ACC, and ACC-r-MTL. It decreased functional connectivity of ACC-visual areas in the occipital lobe.	Increased ACC-MTL connectivity correlated with increased scores on the self-compassion questionnaire.
LSD (50, 51)	14/14	28 ± 4	100 µg, po.	Five participants had previously used a hallucinogen, and 13 participants had previously used a stimulant.	Double-blind, crossover, randomized, placebo-controlled design.	LSD increased functional connectivity between ASM and thalamus, and between SN and thalamus.	ASM-thalamus hyperconnectivity correlates with blissful state, insightfulness, audio-visual synesthesia, and changed meaning of percepts on 11D-ASC.
LSD (52)	10/10	25 ± 4	13 µg, sublingual	Some participants are drug users	Double-blind, crossover, randomized, placebo-controlled design.	LSD increased connectivity of AMG-r-angular gyrus, AMG-r-mFG and AMG-cerebellum. It decreased AMG-postcentral gyrus and AMG-the superior temporal gyrus.	The increase in AMG-mFG connectivity is correlated with positive mood on VAS.
LSD (59)	11/4	30.5 ± 8.0	75 µg LSD, iv.	All participants received LSD at least once	Single-blind, balanced order, crossover, placebo-controlled design.	LSD increased functional connectivity between V1 and cortical and subcortical regions, and connectivity of vmPFC-caudate, vmPFC-inferior FG, PH-dorsal mPFC, and PH-r-DLPFC. LSD decreased connectivity of PH-RSC, PH-PCC, and vmPFC-PCC.	Decreased functional connectivity between PH and RSC correlated with increased VAS ratings of ego-dissolution and altered meaning on the ASC. No correlation is significant.
LSD (24)†	10/10	32 ± 11	100 μg LSD	Two participants had prior experiences with hallucinogenic drugs.	Double-blind, crossover, randomized, placebo-controlled design.	LSD decreased functional connectivity of within the DMN, AUD, sensorimotor networks, and visual networks. LSD increased the connectivity between-RSNs on a whole-brain level (particularly the cerebellum and the ECN).	Not Available
LSD (46)†	10/10	32 ± 11	100 μg LSD, po	Two participants had prior experiences with hallucinogenic drugs.	Double-blind, crossover, randomized, placebo-controlled design.	LSD increased the global functional connectivity especially in thalamus and striatum.	Increased functional connectivity of thalamus-r-Fmg correlates with the VRS on 5D-ASC; Increased connectivity of the thalamus-r-insula correlates with increased score in AA on 5D-ASC.
LSD (49)	19/5	25 ± 3.6	100 µg, po.	Healthy participants	Double-blind, crossover, randomized, placebo-controlled design.	LSD increased the global connectivity, especially in sensory, somatomotor network, and thalamus.	The increased connectivity within somatomotor network is correlated significantly with blissful state, disembodiment, changed meaning of percepts, elementary imagery and spiritual experience on 11D-ASC.
LSD (22)^††^	16/4	30.9 ± 7.8	75 μg, iv.	All participants have had at least one previous experience with a classic psychedelic drug.	Single-blind, >balanced order, crossover, placebo-controlled design.	LSD decreased functional connectivity within the DMN.	Decreased functional connectivity within the DMN is correlated with decreased mental time travel to the past.
LSD (23)^††^	16/4	30.9 ± 7.8	75 μg, iv.	All participants have had at least one previous experience with a classic psychedelic drug.	Single-blind, balanced order, crossover, placebo-controlled design.	LSD increased global connectivity of high-level association cortices (partially overlapping with the DMN, SN, and FPN) and the thalamus.	Increased global connectivity correlates with ego dissolution.
MDMA (50, 51)	14/14	28 ± 4	125 µg, po.	Five participants have used a hallucinogen, and 13 participants have used a stimulant.	Double-blind, crossover, randomized, placebo-controlled design.	MDMA increased functional connectivity of ASM-thalamus and decreased connectivity of SN-thalamus.	Decreased thalamocortical connectivity do not correlate with any of the 5D-ASC subscales.
MDMA (48, 58)^*^	18/7	34 ± 11	100 mg encapsulated MDMA-HCl	All participants have had exposure to MDMA at least once.	Double-blind, crossover, randomized, placebo-controlled design.	MDMA increased connectivity of vmPFC-visual cortex, HIP- dACC, HIP-r- AMG, and HIP-r-mFG, AMG-brainstem and AMG-aPH gyrus. It decreased connectivity of vmPFC-the midbrain, vmPFC-thalamus, vmPFC-AMG, vmPFC-PCC, HIP-mPFC, HIP -left posterior PH/Fmg, HIP-left temporal cortex, AMG-cerebellum, AMG-left temporal cortex, AMG-medial OBC, and AMG-sgACC. MDMA decreased connectivity of visual-sensorimotor networks.	The trends toward decreased functional connectivity of vmPFC-PCC and HIP-vmPFC and increased functional connectivity of AMG-HIP correlated with intense and positive subjective effects were reported, but neither correlation was significant.
MDMA (60)	23/22	26.2 ± 4.4	125 mg MDMA	Some participants were prior drug users	Double-blind, crossover, randomized, placebo-controlled design.	MDMA decreased connectivity within Vis, DMN, the sensorimotor network, and the cerebellar network. MDMA increased connectivity within FPN.	Not Available
MDMA (61)	6/3	41.3 ± 10.9	30, 75 or 125 mg MDMA HCl	Participants with post-traumatic stress disorder	Double-blind, randomized, dose-response trial.	MDMA increased connectivity of AMG-HIP, particularly in the left hemisphere.	Not Available
MDMA (47)^*^	18/7	34 ± 11	100 mg p.o, MDMA-HCl	All participants have had exposure to MDMA at least once.	Double-blind, crossover, randomized, placebo-controlled design.	MDMA decreased FC of r-insula-AI, and r-insula-DLPFC.	Greater trait anxiety is correlated with decreased connectivity between the r-insula-DLPFC; Unusual bodily sensations is correlated decreased FC of the r-insula-DLPFC and the r-insula- aCG.
Psilocybin (56)	10/5	51.3 ± 12.3	0.14 mg/kg, po.	Participants have had long-term meditation practice and been administrated psilocybin before.	Blinded, parallel design, placebo-controlled design.	Psilocybin reduced connectivity of rCLA-AUD, r-CLA-DMN, and left CLA-FPTC. It increased FC of rCLA-FPTC.	Not Available
Psilocybin (45)^#^	13/2	32 ± 8.9	2mg, iv.	All participants received psilocybin at least once.	Crossover, placebo-controlled design.	Psilocybin increased functional connectivity of aDMN-SN, aDMN-r-FPN, aDMN-AUD, aDMN- DAN, and DMN-TPN.	There were suggestions of positive correlations between increased connectivity of DMN-TPN and ratings of psychedelic effects, but no correlation maintained statistical significance following multiple groups comparison correction.
Psilocybin (long-term effect) (62)	11/4	42.8 ± 10.5	0.14, 0.35 mg/kg, 7 d apart	Patients with treatment-resistant depression	Open label study, pre- and post-test design.	Psilocybin increased connectivity of sgACC-PCC and FC of vmPFC-iLPC. It decreased connectivity of PH-LPFC and PH-mPFC.	Not Available
Psilocybin (26)^#^	13/2	32 ± 8.9	2mg, iv.	All participants received psilocybin at least once.	Crossover, placebo-controlled design.	Psilocybin caused a significant decrease in the positive coupling between the mPFC and PCC.	Not Available
Psilocybin (57)	13/5	54.4 ± 13.2	0.14 mg/kg, po.	50% participants had previous experience with a hallucinogen.	Single-blind, crossover, placebo-controlled design.	Psilocybin decreased connectivity of thalamus-Vis, thalamus-DMN and within thalamus.	Decreased connectivity within thalamus correlated to larger decreases in fusion of one’s personal self into larger whole, sense of reverence or sacredness, and feelings of peace and tranquility on MEQ. Decreased connectivity of thalamus-Vis correlates to larger increases in positive emotional valence, overall effect of psilocybin.
Psilocybin (44)^#^	13/2	32 ± 8.9	2mg, iv.	All participants received psilocybin at least once.	Crossover, placebo-controlled design.	Psilocybin decreased connectivity of MTL-high‐level cortical regions, FC within SN, and FC of aPHC- cortical regions.	Ego-dissolution is associated with decreased interhemispheric communication, disintegration of the SN, and disruption of the connection between MTL areas and the parietal lobes.
Psilocybin (63)	6/4	28.3 ± 3.4	0.2–0.3 mg/kg, po.	All participants were healthy and psychedelic-naïve	Open label study, pre- and post-test design., non-randomized, single-blind.	Psilocybin decreased connectivity within ECN 1 week and 3 months after psilocybin.	Decreased connectivity within ECN is associated with psilocybin-induced, long-lasting increases in mindfulness.
Psilocybin (54)	12/11	26.3	0.2 mg/kg, po.	All participants were healthy	Double-blind, randomized, counterbalanced, crossover.	Psilocybin reduced the associative connectivity, increased sensory, global connectivity.	Not Available
Psilocybin (55)	23/15	51.66 ± 8.32	315 μg/kg p.o.	All participants have had meditation practice, and familiarity with longer periods of intensive meditation.	Randomized, double-blind, parallel design, placebo-controlled design.	Psilocybin decreased functional connectivity within the DMN (antero-posterior DMN).	Drug-induced ego dissolution was associated with a decrease FC of antero-posterior DMN.

Superscripts indicate secondary analyses: ^#^psilocybin studies (44) and (45) reanalyzed data from (26); ^†^LSD study (24) reanalyzed data from (46); ^††^LSD studies (22) and (23) share a sample; ^*^ MDMA study (47) reanalyzed data from (48).

PCC, posterior cingulate cortex; YMRS, Young Mania Rating Scale; ACC, anterior cingulate cortex; MTL, medial temporal lobe; SN, salience network; HRS, Hallucinogen Rating Scale; ASM, auditory-sensorimotor network; 11D-ASC, 11 Dimensions Altered States of Consciousness; AMG, amygdala; mFG, middle frontal gyrus; VAS, Visual Analog Scale; V1, primary visual cortex; vmPFC, ventral medial prefrontal cortex; PH, parahippocampus; DLPFC, dorsolateral prefrontal cortex; RSC, retrosplenial cortex; FG, frontal gyrus; AUD, auditory network; ECN, executive control network; Fmg, right fusiform gyrus; VRS, visionary restructuralization; AA, auditory alteration; FPN, frontoparietal network; HIP, hippocampus; dACC, dorsal ACC; aPHC, anterior parahippocampal cortex; sgACC, subgenual ACC; OBC, orbitofrontal cortex; Vis, visual network; AI, anterior insula; FPTC, frontoparietal task control network; CLA, claustrum; TPN, task-positive network; iLPC, infralimbic prefrontal cortex; MEQ, Mystical Experience Questionnaire; aCG, anterior mid-cingulate gyrus.

Two major outcomes were extracted from the papers included in those studies: (i) subjective effects, and (ii) functional connectivity. We also summarized the results of studies that reported the correlation between the subjective effects and functional connectivity, displayed in **Table 3**.

### Subjective effects of psychedelics

Research on psychedelics consistently highlights the profound subjective effects they induce, with different studies utilizing a range of scales to quantify these experiences. In ayahuasca research, subjective effects were assessed using the Young Mania Rating Scale (YMRS) and Hallucinogen Rating Scale (HRS). For LSD studies, both 5-dimension altered states of consciousness (5D-ASC) and 11D-ASC were employed. Psilocybin research utilized the 5D-ASC, mystical experience questionnaire (MEQ), mindful attention awareness scale (MAAS) and visual analog scale (VAS), while MDMA studies made use of the 5D-ASC, VAS, MEQ, and Adjective Mood Rating Scale (AMRS). YMRS was used to measure mania symptoms of ayahuasca intake. HRS evaluates the subjective effects of hallucinogens. 5D-ASC and 11D-ASC evaluate altered states of consciousness (64). MEQ assesses mystical or profound personal experiences (64). MAAS measures mindfulness, focusing on how attentively and openly individuals observe their current experiences (65). VAS is a measurement of pain (66), and the AMRS is a self-report questionnaire that assesses mood states (67). All subscales of these scales and significant findings on the subjective effects of psychedelics are shown in **Table 2**.

Ayahuasca has a positive effect on elevated mood and an increase in energy or motivation via the YMRS and induces a spectrum of hallucinatory experiences, as evidenced by the HRS. However, there are contrasting findings regarding ayahuasca’s effect on volition assessed by HRS between studies, potentially due to differences in experimental design, or doses. However, studies of LSD and psilocybin more focused on the altered state of consciousness. LSD has a pronounced impact on altered states of consciousness, mystical experiences, and the intensity of subjective perceptions, though some variances exist, particularly concerning specific dimensions in the 5D-ASC. Psilocybin also influences altered states of consciousness, mystical experiences, perceptual shifts, and longer-term traits like openness and mindfulness. Although studies of MDMA did not report a significant difference between 5D-ASC, participants frequently expressed intense sensations of joy, a sense of well-being, and a feeling of connectedness, supported by the high ratings in “I felt amazing” after the MDMA treatment.

### Functional connectivity alterations induced by psychedelics

Psychedelics have garnered significant attention in the realms of neuroscience and clinical medicine due to their potential therapeutic effects. The alterations they induce in brain functional connectivity are one of the core mechanisms underlying their actions. To provide a concise overview of the functional connectivity changes induced by ayahuasca, LSD, psilocybin and MDMA, we collated key findings from various studies in **Table 3**, column 7.

These studies elucidate the complex acute effects that different psychedelics have on brain functional connectivity. Acute ayahuasca intake was found to decrease functional connectivity within the PCC/Precuneus of the DMN relative to pre-intake levels (32). Additionally, subacute ayahuasca intake has been shown to similarly decrease functional connectivity within PCC of the DMN a day post-intake versus placebo, with concomitant increases in functional connectivity within ACC of the SN, and between the SN and DMN (31). Moreover, increased connectivity between the ACC and right medial temporal lobe (MTL), as well as between PCC and ACC, was reported in another acute ayahuasca study (33), These findings are consistent with those reported by *Pasquini L* (31) and *Palhano-Fontes F* (32), who observed similar patterns in their respective studies.

LSD research presents a wide array of effects on the functional connectivity of the brain. A consistent observation across studies is the increase in global connectivity post-acute LSD administration, as compared to placebo (24). However, this trend was predominantly noted as increased functional connectivity within sensory and somatomotor regions, specifically within the occipital cortex, superior temporal gyrus, and postcentral gyrus (49). These findings were supported by the other studies, where acute LSD-induced functional connectivity increases between the primary visual cortex (V1) and other brain areas were observed in a visual cortex seed-based study (59), and auditory-sensorimotor-thalamic hyperconnectivity was reported in individuals after LSD administration compared to placebo (50). Furthermore, several seed-based studies have highlighted significant functional connectivity changes involving the thalamus, vmPFC, parahippocampus (PH), and amygdala (50, 52, 59). Specifically, LSD increased thalamocortical connectivity and functional connectivity within the SN (50). Additionally, increased functional connectivity was observed from the PH to both the dorsal mPFC and the right dorsolateral PFC (r-DLPFC) and from the vmPFC to the bilateral caudate and inferior frontal gyrus. Conversely, there was a decrease in functional connectivity from the PH to the retrosplenial cortex (RSC) and PCC, and from the vmPFC to the PCC (59). A microdose of LSD (13 micrograms) was also shown to alter the functional connectivity of the brain. The study revealed increased functional connectivity between the amygdala and regions such as the right angular gyrus, right middle frontal gyrus (mFG), and the cerebellum but decreased functional connectivity between the amygdala and both the left and right postcentral gyrus and the superior temporal gyrus compared with placebo (52).

Given the intricate network alterations and the focus on specific brain regions by various researchers, a study has been conducted to clarify how LSD acutely impacts functional connectivity, by separately examining changes within- and between-networks (49). Decreases in Functional connectivity within the DMN, auditory-sensorimotor networks (ASM), and visual networks were noted, while there was an increase in functional connectivity between the resting-state networks at the whole-brain level, specifically involving the thalamus and striatum. The within-network changes align with those reported in ayahuasca studies (31, 32). Additionally, another study summarized that LSD-induced hyper-connectivity was predominantly observed in sensory and somatomotor areas, whereas hypo-connectivity was observed across subcortical regions (apart from the amygdala and sensory thalamus) and cortical areas tied to associative networks, such as the medial and lateral prefrontal cortex, the cingulum, the insula, and the temporoparietal junction (49), consistent with previous findings (50, 52, 59).

Similar to LSD, MDMA decreased functional connectivity within two networks, the DMN, and the sensorimotor network (60), and elicited auditory-sensorimotor–thalamic hyperconnectivity compared with placebo. However, distinct from LSD, MDMA induced hypoconnectivity within the SN and this change was also observed in PTSD patients two months post-MDMA intake, compared to before-intake levels (47, 50). Beyond these within-network changes, MDMA also acutely affected between-network connectivity, notably inducing hypoconnectivity between midline cortical regions, including the mPFC and MTL regions. This was evidenced by decreased functional connectivity between the vmPFC and midline cortical regions, thalamus, AMG, and PCC, as shown through a seed-based analysis involving the vmPFC, hippocampus (HIP), and AMG. Decreased functional connectivity of HIP with mPFC, left posterior PH/fusiform gyrus (Fmg), left temporal cortex, and decreased functional connectivity of AMG with cerebellum, left temporal cortex, medial orbitofrontal cortex (OBC), and AMG-subgenual ACC (sgACC) were also observed in the study. Conversely, MDMA increased connectivity in certain areas, including the functional connectivity from the vmPFC to the visual cortex, the HIP to the dACC, r-AMG, and r-mFG, as well as from the AMG to the brainstem and anterior parahippocampal gyrus (PHG) (48, 58).

Similar to the other psychedelics mentioned above, the alterations of functional connectivity of DMN, mPFC and MTL were also observed in the studies of psilocybin. Specifically, the study showed a decrease in connectivity within associative networks-important for information storage and organization in the brain (63)-as well as reduced functional connectivity within the DMN (54), SN (57), between the mPFC and the PCC (26), and between the MTL and higher-level cortical areas (57). In addition, the acute study of psilocybin reported identified the involvement of a novel brain structure, the claustrum (CLA), which has not been discussed in previous studies. Psilocybin decreased functional connectivity between the r-CLA and both the auditory network and the DMN, and between the l-CLA and the frontoparietal task control network (FPTC) (56). A long-term study compared the pre- and post-psilocybin effects on the functional connectivity of the brain and revealed the functional connectivity of executive control network (ECN) was also decreased 1 week after intake (44). On the other hand, psilocybin significantly enhanced global connectivity (63), particularly in thalamocortical (26) and sensory connectivity, compared to placebo. Additionally, it also increased functional connectivity between DMN and TPN (45) and between r-CLA with the FPTC (56). Notably, a long-term study of psilocybin on patients with TRD showed increased functional connectivity within DMN and between vmPFC and bilateral inferior lateral parietal cortex, and consistent decreased in functional connectivity in PH-PFC, 5 weeks after psilocybin intake compared to the baseline level before intake (62).

While ayahuasca, LSD, MDMA, and psilocybin are associated with changes in brain connectivity, such as alterations in the DMN and enhanced sensory and thalamocortical connectivity, the precise relationship between these functional connectivity changes and specific therapeutic outcomes remains an area of active research. Further studies are needed to clarify how these connectivity changes may contribute to the observed psychological effects and their potential therapeutic applications.

### The relationship between functional connectivity alterations and subjective effects of psychedelics

Analyzing the interplay between functional connectivity alterations and the subjective effects of psychedelics may offer a greater understanding of their neuropsychological impact. The details of each study are shown in **Table 3**, column 8.

Enhanced functional connectivity within the SN is associated with a higher somesthesia score, whereas reduced functional connectivity within the DMN is linked to a higher volition score. Additionally, increased connectivity between SN and DMN is related to an elevated affect score, all measured using the HRS scale (31). Somesthesia refers to the assessment of bodily sensations, volition to the ability to interact with oneself, and affect to changes in comfort and emotional state (31). Studies suggest that changes in the DMN are associated with the altered state of consciousness induced by ayahuasca. These changes in DMN connectivity are correlated with increases in YMRS scores (32). Additionally, increased connectivity between the ACC and MTL is associated with higher self-compassion scores (33). It appears that the subjective effects of ayahuasca are regulated through changes in the DMN, SN, and MTL networks.

Research has established a correlation between the DMN and the subjective effects of LSD, highlighting that decreased functional connectivity within the DMN correlates with a lower frequency of mental time travel to the past. This reduction is further associated with the overall intensity of the subjective experiences elicited by LSD (22). Additionally, there is evidence that increased global connectivity under the influence of LSD corresponds with subjective reports of ego dissolution, suggesting a fundamental change in self-perception and consciousness (23). Further analysis reveals that the functional connectivity between the thalamus and specific cortical regions, such as the r-FG and the r-insula, positively correlates with the subjective effects of visionary restructuralization (VRS) and auditory alterations, respectively, on the 5D-ASC scale (46). This is complemented by findings that thalamocortical hyperconnectivity is associated with a range of altered states, including blissful states, insightfulness, audio-visual synesthesia, and a shift in the perceived meaning of percepts, as measured by the 11D-ASC (50). Moreover, increased connectivity within the somatomotor network has been significantly linked to blissful states, disembodiment, altered perception of meanings, elementary imagery, and spiritual experiences (52). Even at low doses, LSD has been shown to increase connectivity between the AMG and mFG, correlating with positive mood enhancements (52). However, the anticipated correlation between decreased connectivity from the PH to the RSC and experiences of ego dissolution and altered meanings did not achieve statistical significance (59). Like ayahuasca, these findings collectively highlight LSD’s capacity to modulate subjective experiences through its differential effects on the DMN, SN, MTL (PH and AMG). However, subjective effects of LSD also involve alterations in the thalamus and thalamocortical networks (46).

MDMA decreased thalamocortical connectivity, similar to LSD, but this change does not correlate with any of the 5D-ASC subscales (50). Besides, the trends toward decreased functional connectivity of vmPFC-PCC and HIP-vmPFC and increased functional connectivity of AMG-HIP correlate with intense and positive subjective effects were reported, but neither correlation was significant (48). An interesting study reported that subjective effects of acute MDMA, including trait anxiety measured by the Spielberger State-Trait Anxiety Inventory (STAI) and unusual bodily sensations, correlated with decreased functional connectivity. Specifically, reductions were observed between the right insula (r-insula) and the dorsolateral prefrontal cortex (DLPFC), as well as between the r-insula and the anterior mid-cingulate gyrus (aCG) (47). Notably, the altered bodily sensations mentioned above were viewed as an indicator of dysregulated introspection. Moreover, a suggested link between interoceptive processing and trait anxiety suggests that the introspection structure could be a contributing factor to anxiety vulnerability (47).

Although many studies have reported functional changes caused by psilocybin, including in the DMN, SN, ECN, thalamus, and MTL, the relationship between subjective effects and these functional changes induced by psilocybin remains complex. While positive correlations between functional connectivity of DMN-TPN and ratings of psychedelic effects were reported, no correlation maintained statistical significance following multiple groups comparison correction (45). However, decreased connectivity within DMN (mPFC-PCC) is associated with increased intensity of the subjective effects (26). Also, a decrease in functional connectivity of antero-posterior DMN is correlated with drug-induced ego dissolution (55). Drug-induced ego-dissolution was also associated with decreased interhemispheric communication, decreased functional connectivity within SN, and functional connectivity between MTL areas and the parietal lobes (44). Moreover, decreased functional connectivity within ECN is associated with psilocybin-induced, long-lasting increases in mindfulness in participants with the experience of mindfulness (63). A study investigated the changes in within-network and between-network connectivity related to the thalamus induced by psilocybin. Decreased functional connectivity within thalamus correlated to larger decreases in the fusion of one’s personal self into a larger whole, a sense of reverence or sacredness, and feelings of peace and tranquillity on MEQ (57). Decreased functional connectivity of thalamus-Vis correlates to increases in a score of positive emotional valence, and the overall subjective effect of psilocybin (57).

These results suggest that the subjective effects of psychedelic drugs are associated with decreased connectivity within DMN, increased global connectivity, and complicated changes in some critical networks like SN, thalamocortical networks, or key hubs, such as insula, AMG, mPFC and PCC, enabling a state of unconstrained cognition.

## Discussion

Psychedelic-assisted psychotherapy has shown promise in treating a variety of mental health disorders, particularly in addressing MDD, PTSD, and anxiety associated with life-threatening illnesses (68). Research indicates that, within therapeutic settings, LSD significantly reduces anxiety in patients with life-threatening conditions (69), while psilocybin and MDMA have demonstrated rapid antidepressant effects (9, 70, 71). The subjective effects of psychedelics are an area of active inquiry that merits further attention. Insights into the brain network changes associated with these effects could prove valuable for designing and customizing future therapeutic interventions for clinical patients using psychedelics. The subjective effects induced by psychedelics include alterations in states of consciousness, mystical experiences, and enhanced affective cognition. In healthy subjects, LSD and psilocybin significantly affect changes in states of consciousness, while MDMA induces intense feelings of joy, well-being, and connectedness. Here, we review the relationship between changes in brain functional connectivity and subjective effects, highlighting functional networks commonly reported in relation to subjective effects of these substances, which include the DMN, SN, thalamocortical pathways, and the alterations in functional connectivity between them. Notably, most current research focuses on healthy participants.

### The roles of DMN, SN, thalamus in subjective effects of psychedelics

**Table 3** visualizes the correlation between the functional connectivity and subjective effects of psychedelics. Research suggests that psychedelics, including ayahuasca, LSD, MDMA, and psilocybin, lead to substantial alterations in brain functional connectivity. These changes predominantly impact the DMN, the SN, and thalamocortical pathways. These connectivity shifts closely correlate with subjective effects reported during psychedelic state, such as altered states of consciousness, mystical experiences, and shifts in perception and emotion (summarized in **Table 4**).

**Table 4:** The summary of the brain networks and associated subjective effects of psychedelics.

Networks		Subjective effects
DMN (mPFC, PCC/precuneus, RSC)	within DMN	volition (31), altered state of consciousness (32), decreased mental time travel to the past (22), ego-dissolution (55).
	between DMN and the other regions	affect (31), ego-dissolution and altered meaning (59).
SN (AI, ACC, AMG)	within SN	somesthesia (31), ego-dissolution (44).
	between SN and the other regions	affect (31), self-compassion (33), positive mood (52), auditory alteration (46), trait anxiety (47).
Thalamus	within thalamus	fusion of one’s personal self into larger whole, sense of reverence or sacredness, and feelings of peace and tranquility (57).
	between thalamus and the other regions	blissful state, insightfulness, audio-visual synesthesia, and changed meaning of percepts (50, 51), visionary restructuralization (46), positive emotional valence (57).

mPFC, medial prefrontal cortex; PCC, posterior cingulate cortex; RSC, retrosplenial cortex; DMN, default mode network; SN, salience network; AI, anterior insula; ACC, anterior cingulate cortex; AMG, amygdala.

Subjective effects is followed by a number indicating its reference. Except for a decrease noted in one scale, all other scales showed increased scores.

One consistent finding is the observation across different substances of reduced functional connectivity within the DMN, a network implicated in self-referential thought processes. Decreased functional connectivity within DMN may represent a common neural mechanism through which psychedelics elicit subjective effects associated with potential therapeutic efficacy, potentially by reducing maladaptive rumination and fostering a cognitive environment conducive to restructuring. The vmPFC, an integral component of the DMN, is instrumental in modulating emotional processes, a role that has been observed in mechanisms such as the extinction of conditioned fear and the deliberate modulation of negative emotions. Koenigs et al. have suggested that the contribution of vmPFC to PTSD may be attributed to its functions in self-awareness and introspection (72, 73). A reduced ability for self-awareness or introspection is associated with lessening the central symptoms of PTSD as distress and anxiety related to previous personal experiences. PCC, another region of DMN connected with the hippocampal memory system and the OBC, is involved in reward and emotion processing. Patients with MDD have shown increased functional connectivity between PCC and OBC, which might contribute to the rumination about sad memories and events in depression (74).

Moreover, the increased thalamocortical connectivity observed with substances like LSD and psilocybin may underpin their capacity to induce profound shifts in perception and consciousness (50, 51, 75). This alteration could facilitate a ‘reset’ of sensory and cognitive processing, enabling a more direct and unfiltered experience of the world. Such effects may contribute to reported therapeutic benefits, including increased mindfulness and the resolution of traumatic memories. Additionally, by modulating the SN, psychedelics might potentiate psychotherapy by intensifying or reshaping the importance of specific experiences or perceptions (76) and increasing cognitive flexibility, leading to novel thoughts or patterns of thinking (77), which are beneficial for depression treatment.

LSD is renowned for inducing vivid and profoundly symbolic experiences, including somatic sensations, archetypal encounters reminiscent of Jungian themes, and rich symbolisms. The observed enhancement of interconnectivity within the brain may be linked to an increased capacity for engaging with symbolic and dream-like states that are typically inaccessible during normal waking consciousness. This altered connectivity could offer an alternative pathway for the mind to revisit emotionally charged material that remains otherwise elusive (49). As previously discussed, both psilocybin and LSD globally increase functional connectivity. This heightened connectivity may reduce reliance on learned patterns and foster a more intuitive state. The concept of a ‘liberated state’ refers to a psychological openness that allows individuals to explore new perspectives and emotions, potentially aiding the processing and integration of traumatic memories during therapeutic sessions (49, 63).

MDMA, while decreasing functional connectivity within the DMN, does not exhibit the same increase in global connectivity noted in classic psychedelics. This distinction may suggest a unique mode of action for MDMA compared to LSD and psilocybin, which not only decreases DMN connectivity but also induces an increase in global integration of the brain. In addition, MDMA’s influence on the sensorimotor network appears to primarily manifest as a decrease in connectivity. The decrease might be linked to the distinct empathogenic effects of MDMA, which could influence the processing of emotions and sensations. On the other hand, classic psychedelics like LSD and psilocybin have shown both increases and decreases in sensorimotor connectivity. This variability could contribute to the diverse range of sensory and perceptual changes experienced with these substances. While effects of MDMA on functional connectivity, notably within the DMN, consistent with changes observed with classical psychedelics, the direct correlation to subjective effects is less reported. Unlike LSD and psilocybin which showed increased connectivity within SN, MDMA decreased connectivity within SN. This difference might indicate differences in how these substances modulate attention and the integration of emotional and sensory stimuli which are associated with SN. These differences in network modulation by MDMA, as opposed to other psychedelics, underscore the importance of considering each substance’s unique neural signature when evaluating their therapeutic potential and subjective effects. Additionally, alterations in functional connectivity related to the AMG have been documented, aligning with MDMA’s potential role in fear extinction for PTSD patients. Given the remarkable effects of MDMA in treating PTSD, further investigation into the connectivity changes within influential brain regions-such as PFC, HIP, and AMG-and their relationship to symptom reduction justifies continued exploration.

The functional changes caused by psychedelic drugs are not changes in a single brain region or a single network. In terms of research direction, it may be beneficial to systematically categorize networks showing increased or decreased connectivity and then assess these changes in the context of overall network structure of the brain to gain a clearer understanding of the drugs’ effects. Clarifying the relationship between hyperconnectivity networks and hypoconnectivity networks, and whether the connections of these networks can transition between hyperconnectivity networks and hypoconnectivity, is crucial for understanding how information flows in these complex networks and how the flow of information is reflected in subjective effects.

In conclusion, the influences of these substances seem to be connected to the personal experiences they induce, which are thought to be tied to significant shifts in brain communication patterns, like DMN, SN, and thalamocortical connectivity. These shifts might be a shared way these substances work, possibly creating a mental state open to change and leading to profound alterations in perception and self-awareness. However, a detailed understanding of the specific neural mechanisms and subjective effects associated with psychedelic-assisted psychotherapy is essential for optimizing clinical application. Consequently, further research is essential to extend the findings from healthy participants’ and patients’ responses in clinical settings.

## Limitations

In this review, our examination of psychedelic substances is focused on those with well-documented neuropharmacological profiles and demonstrated therapeutic potential. Future work may broaden the scope of this review to include a more comprehensive array of psychedelics, deepening our understanding of their potential therapeutic mechanisms. Furthermore, it should be noted that the sample sizes in these studies were relatively modest, with instances of sample reuse observed in some cases. This aspect warrants attention in terms of the reproducibility of the findings, particularly given the potential for individual differences to significantly impact outcomes within the psychedelics field. It would be beneficial for future research to aim for larger and more diverse participant pools to strengthen the reliability and broader relevance of the results.

Most psychedelic research, including the studies analyzed in this review, has been conducted on healthy individuals. While such studies are invaluable for understanding the basic mechanisms by which psychedelics affect brain function and subjective experience, they inherently limit our ability to extrapolate findings to clinical populations. Individuals with psychiatric disorders often exhibit alterations of functional connectivity that possibly have impacts on their response to psychedelics. For example, changed functional connectivity within DMN has been reported in individuals with depression. The impact of psychedelics on the DMN in healthy individuals may not accurately reflect the therapeutic potential or risks for those with clinical populations. Moreover, the subjective effects of psychedelics, such as ego dissolution or emotional breakthroughs, may manifest differently in clinical populations since they might have distinct baseline experiences of self-concept and emotional regulation, highly likely in individuals who have dissociative symptoms.

Considering the challenges of conducting research with clinical populations, the logistics of conducting research within a clinical setting are often more complex. In addition, studies on healthy volunteers tend to focus on the acute neural and psychological effects of psychedelics, which may not correspond to long-term therapeutic outcomes in clinical populations. Therefore, the progress from acute subjective and neural changes to sustained clinical improvement remains unclear. Future research must include clinical populations to substantiate the therapeutic efficacy of psychedelics, considering the distinct neurobiological and psychological states of mental health disorders.

## Author contributions

ZY: Writing – original draft, Writing – review & editing, Conceptualization, Formal analysis, Investigation, Methodology, Project administration, Data curation. LB: Writing – original draft, Writing – review & editing. OW: Writing – original draft, Writing – review & editing. LX: Writing – original draft, Writing – review & editing. LD: Writing – original draft, Writing – review & editing. EV: Writing – original draft, Writing – review & editing. AG: Writing – original draft, Writing – review & editing. X-ML: Writing – original draft, Writing – review & editing. MM: Writing – original draft, Writing – review & editing. FW: Writing – original draft, Writing – review & editing. BC: Writing – original draft, Writing – review & editing. IW: Writing – original draft, Writing – review & editing. YZ: Conceptualization, Methodology, Supervision, Writing – original draft, Writing – review & editing. AWC: Conceptualization, Methodology, Supervision, Writing – original draft, Writing – review & editing.
