# Supplementary material for: Alterations in brain network connectivity and subjective experience induced by psychedelics: a scoping review
Source: Front Psychiatry. 2024 May 14;15:1386321. doi: 10.3389/fpsyt.2024.1386321 (PMC11131165; doi:10.3389/fpsyt.2024.1386321)
Supplement: Supplementary file 1 [file Table_1.docx]

Searching strategy

**Ovid MEDLINE(R) ALL 1946 to Jun 26, 2023**

Date searched: Jun 6, 2023

Number of results: 223

1. 3,4-methylenedioxyamphetamine/ or lysergic acid diethylamide/ or mescaline/ or n,n-dimethyltryptamine/ or n-methyl-3,4-methylenedioxyamphetamine/ or psilocybin/

2. (psychedelic* or "serotonin 2A receptor agonist*" or DMT or psilocyb* or psilocib* or ayahuasca or ayawasca or entactogen* or mdma or methylenedioxyamphetamine or ecstasy or mescalin* or magic mushroom* or psilocin or lysergic acid diethylamide or lsd).mp.

3. (((brain or network or functional or global or default or neural) adj4 (connectivity or disconnectivity)) or functional reorgani?ation or functional organization or (integrity adj5 network) or Fmri or functional mri or functional magnetic resonance imaging or bold imaging or bold functional imaging or blood-oxygen-level-dependent-imaging).mp.

4. (1 or 2) and 3 (223 results)

**Embase 1974 to to 2023 Jun 26 (OVID Interface)**

Date searched: Jun 6, 2023

Number of results: 408

1. psychedelic agent/ or lysergic acid/ or lysergide/ or mescaline/ or "n (3,4,5 trimethoxyphenylethyl)aziridine"/ or n,n diisopropyl 5 methoxytryptamine/ or psilocin/ or psilocybine/

2. psychedelic therapy/

3. (psychedelic* or "serotonin 2A receptor agonist*" or DMT or psilocyb* or psilocib* or ayahuasca or ayawasca or entactogen* or mdma or methylenedioxyamphetamine or ectstasy or mescalin* or magic mushroom* or psilocin or lysergic acid diethylamide or lsd).mp.

4. 1 or 2 or 3

5. functional magnetic resonance imaging/

6. functional connectivity/

7. (((brain or network or functional or global or default or neural) adj4 (connectivity or disconnectivity)) or functional reorgani?ation or functional organization or (integrity adj5 network) or Fmri or functional mri or functional magnetic resonance imaging or bold imaging or bold functional imaging or blood-oxygen-level-dependent-imaging).mp.

8. 5 or 6 or 7

9. 4 and 8

10. limit 9 to conference abstracts

11. 9 not 10

**APA PsycInfo 1806 to Jun 26 2023 (OVID Interface)**

Date searched: Jun 26, 2023

Number of results: 151

1. psychedelic experiences/

2. methylenedioxymethamphetamine/

3. hallucinogenic drugs/ or lysergic acid diethylamide/ or mescaline/ or psilocybin/

4. (psychedelic* or "serotonin 2A receptor agonist*" or DMT or psilocyb* or psilocib* or ayahuasca or ayawasca or entactogen* or mdma or methylenedioxyamphetamine or ecstasy or mescalin* or magic mushroom* or psilocin or lysergic acid diethylamide or lsd).mp.

5. or/1-4

6. functional magnetic resonance imaging/

7. brain connectivity/

8. (((brain or network or functional or global or default or neural) adj4 (connectivity or disconnectivity)) or functional reorgani?ation or functional organization or (integrity adj5 network) or Fmri or functional mri or functional magnetic resonance imaging or bold imaging or bold functional imaging or blood-oxygen-level-dependent-imaging).mp.

9. 6 or 7 or 8

10. 5 and 9

**Scopus (advanced search)**

Date searched: Jun 26, 2023

Number of results: 418 results

TITLE-ABS-KEY(psychedelic* or "serotonin 2A receptor agonist*" or DMT or psilocyb* or psilocib* or ayahuasca or ayawasca or entactogen* or mdma or methylenedioxyamphetamine or ecstasy or mescalin* or "magic mushroom*" or psilocin or "lysergic acid diethylamide" or lsd) AND TITLE-ABS-KEY(((brain or network or functional or global or default or neural) W/4 (connectivity or disconnectivity)) or "functional reorganization" or "functional organisation" or "functional organization" or "functional organisation" or (integrity W/5 network) or Fmri or "functional mri" or "functional magnetic resonance imaging" or "bold imaging" or "bold functional imaging" or blood-oxygen-level-dependent-imaging)
